# Quantitative Electroencephalography Analysis for Improved Assessment of Consciousness Levels in Deep Coma Patients Using a Proposed Stimulus Stage

**DOI:** 10.3390/diagnostics13081383

**Published:** 2023-04-10

**Authors:** Çiğdem Gülüzar Altıntop, Fatma Latifoğlu, Aynur Karayol Akın, Ayşe Ülgey

**Affiliations:** 1 Department of Biomedical Engineering, Erciyes University, Kayseri 38039, Turkey; 2 Department of Anesthesiology and Reanimation, Erciyes University, Kayseri 38039, Turkey

**Keywords:** Glasgow Coma Scale, consciousness level, electroencephalography, EEG, machine learning, classification, data imbalance

## Abstract

“Coma” is defined as an inability to obey commands, to speak, or to open the eyes. So, a coma is a state of unarousable unconsciousness. In a clinical setting, the ability to respond to a command is often used to infer consciousness. Evaluation of the patient’s level of consciousness (LeOC) is important for neurological evaluation. The Glasgow Coma Scale (GCS) is the most widely used and popular scoring system for neurological evaluation and is used to assess a patient’s level of consciousness. The aim of this study is the evaluation of GCSs with an objective approach based on numerical results. So, EEG signals were recorded from 39 patients in a coma state with a new procedure proposed by us in a deep coma state (GCS: between 3 and 8). The EEG signals were divided into four sub-bands as alpha, beta, delta, and theta, and their power spectral density was calculated. As a result of power spectral analysis, 10 different features were extracted from EEG signals in the time and frequency domains. The features were statistically analyzed to differentiate the different LeOC and to relate with the GCS. Additionally, some machine learning algorithms have been used to measure the performance of the features for distinguishing patients with different GCSs in a deep coma. This study demonstrated that GCS 3 and GCS 8 patients were classified from other levels of consciousness in terms of decreased theta activity. To the best of our knowledge, this is the first study to classify patients in a deep coma (GCS between 3 and 8) with 96.44% classification performance.

## 1. Introduction

**Figure 1:**
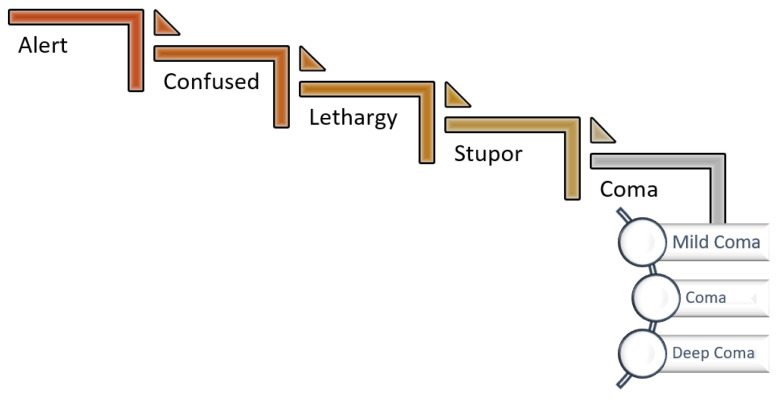
Altered level of consciousness.

Intensive care is defined as all the methods applied to treat disease and to ensure patient survival until partial or complete loss of organ or system functions cause negative effects. Intensive care units (ICUs) are special units that provide the necessary treatment and care to patients who require intensive care. Many patients in the ICU are unconscious due to an organic disorder or sedation. Consciousness is defined as being aware of oneself and one’s environment and adapting to new stimuli. Assessment of the level of consciousness (LeOC) is a significant task that affects stages such as nursing and treatment of the patient. As the patient’s assistance is required during the evaluation of the LeOC, motor and sensory evaluations cannot be made for patients who cannot respond to commands [1,2]. Evaluating the LeOC and coma outcome prediction is very important for medical practice. The LeOC is not a phenomenon directly measured. In the evaluation of consciousness, firstly, verbal stimuli are given to the patient. The motor response for LeOC assessment is evaluated with intense (not harmful) painful stimuli [2,3]. Consciousness levels are shown in Figure 1. Two main factors can be mentioned for consciousness: the level of arousal or wakefulness and the awareness (i.e., the content of consciousness). Impairment of arousal is a process that progresses from lethargy to stupor and then to coma. Coma is the inability of the patient to be aroused. In order to interpret that the patient is in a coma, their eyes must be closed, not responding to environmental stimuli, and not having spontaneous movements [2].

The severity of the disease is analyzed by grading the symptoms and by evaluating many physiological findings. Knowing the severity of the disease is important in terms of devising treatment, estimating the outcomes from the patient, and providing better intensive care. Scoring systems are not the main task of the treatment, but they are helpful in clinical decisions and in reducing hospital costs. Incorrect determination of the severity of the disease causes loss in cost and time, misdiagnosis, and wrong treatment [3,4]. The severity of the disease can be determined by various methods. While the width of the injury is anatomically determined by the injury severity scoring (ISS) method in trauma patients, the Glasgow Coma Scale (GCS) is the most commonly used in determining neurological damage and is an accepted 15-point score used to measure coma [5]. The GCS is a method for providing a simple and reproducible method of assessment of LeOC [6]. The GCS is scored according to the best response of the patients in each of its three categories: eye responses (E), verbal responses (V), and motor responses (M) [7]. The summation of the individual score (E + V + M) classifies the patients into mild closed head injury (score = 13–15); moderate head injury (score = 9–12); severe brain injury, also coma state (score = 3–8); and vegetative state (score < 3) [8]. Many physicians regard a maximum GCS score of 8 as the limit for coma [6]. It is stated that traumatic brain injuries are mainly determined by GCS; however, it is known that GCS is not sufficient and reliable for use by inexperienced clinicians.

Namiki et al. show how the GCS can be misinterpreted by an inexperienced physician. A new graduate assistant was asked to examine the eye, verbal, and motor responses of a patient and to perform a GCS assessment. The GCS assessment was tested with eight different levels of consciousness. As a result of this study, on average, 26 ± 18% of examinees did not provide an accurate evaluation for the eight levels of consciousness selected. The GCS factors, mostly misinterpreted, were “evaluation of mixed speech” and “withdrawal motor response” [9].

It is considered that GCS is measured reliably by trained healthcare personnel [9,10]. However, GCS reliability has been reported to be insufficient in clinical practice [9,10,11,12,13,14] because GCS scoring errors have rarely been investigated [11] and methods that can help improve the accuracy of GCS are very rare [9].

The GCS is the chiefly used score in the evaluation of the neurological condition in the ICU. The GCS has a few limitations; most importantly, it requires an interactive patient, but patients in a coma state (GCS ≤ 8) are unlikely to be active. In the literature, the error rate resulting from evaluation with GCS is stated as 40% [15]. It is not correct to use the scoring systems in patients with impaired motor responses or who have an attention deficit, a lack of arousal, or a lack of perception [16,17]. The GCS is designed for serial assessments, so it should be emphasized that any confidence in predicting outcomes cannot be sufficiently established until a trend is achieved in the GCS [17].

In the statistical analysis of the data obtained from intensive care units, some studies specifically aiming to produce predictive models have been conducted on heart rate, blood pressure, etc. There are studies to automatically detect abnormality in such situations. Automatic estimates are made to eliminate the need for human surveillance [18,19,20]. Monitoring Electroencephalogram (EEG) in ICUs ensures that changes in the neural activities of the brain are noticed immediately and have long-term follow-up. Electroencephalography is a useful tool for noninvasively quantifying neurological function [21].

Gills et al. mention the time and frequency of methods used in the study of the EEG data of patients in the ICU. They extracted autoregression coefficients from EEG data as a feature and examined the forces in frequency bands [22]. Shah et al. obtained the amplitude, autoregression coefficients, and frequency-weighted energy values from the EEG signals belonging to patients in the ICU and classified them using clustering methods [23].

EEG is used to identify sleep disorders, coma, encephalopathy, and brain death [24]. Diagnostic applications are mostly aimed at examining the spectral content of neural oscillations in EEG waves. EEG is used frequently because it is non-invasive and has a high time resolution. EEG is widely used compared with other brain imaging techniques in applications where continuous monitoring of the brain is important [25,26]. Additionally, the creation of a real-time health monitoring system for stroke prediction [27], post-stroke recovery prediction [28], and stroke prognostics [29] using quantitative EEG analysis and machine learning approaches are indicators of the broad scope of use of EEG.

Since the evaluation of consciousness disorders is performed based on behavioral scales, it makes the diagnosis of these patients difficult. A patient in a vegetative state/unresponsive wakefulness syndrome (VS/UWS) is awake but unaware of himself or his environment. A patient in a minimally conscious state (MCS) has awareness of themselves or their environment to some extent. A comatose patient has neither spontaneous alertness nor an eye-opening caused by impulse [30]. Therefore, studies focus on EEG in order to differentiate disorders of consciousness (DOC) patients. Mikola et al. analyzed the EEG recordings to show the correlation of delirium with EEG. The relative powers of the EEG signal were computed for delirium and control groups. They showed that the relative power of EEG signals with a frequency above 8 Hz was lower in the state of delirium. This was pursued at the parietal and central lobes of the brain [31]. Lechinger et al. [32] analyzed various EEG parameters to find an association between EEG signals and Coma Recovery Scale-Revised (CRS-R) score. They showed a positive correlation between the CRS-R score and ratios calculated between frequencies above 8 Hz and frequencies below 8 Hz using Pearson correlation and repeated measures ANOVAs. Piarulli et al. [33] found higher theta/alpha and lower delta power in MCS patients compared with VS/UWS patients. Additionally, Naro et al. [34] revealed lower alpha power in UWS and MCS patients. Kotchoubey et al. [35] showed that conscious patients have indicated much more electrophysiological brain activity than patients in the persistent vegetative state (PVS) and MCS patients. Khanmohammadi et al. [36] obtained a positive relationship between the Intrinsic Network Reactivity Index and GCS. They revealed significant differences between various LeOC. They concluded that power analysis of EEG signals did not give any specific patterns correlated to GCS. There are studies that have shown that a 40 Hz auditory steady-state response (ASSR) as an auditory stimulus may be associated with the state of consciousness of patients in a coma [37,38]. At present, 40 Hz ASSR has been applied for coma outcome prediction [39,40]. Wieser et al. [16] conducted linear backward regression analysis in their work and concluded that 13 variables obtained from the data of 8 patients were adequate to define 74.7% of the variability. The electrocardiogram, P300, and blood pressure signal generated most of the variability in their regression model.

In our study, EEG data were recorded from 39 comatose patients with a new procedure proposed by us. To the best of our knowledge, there is no study to classify the GCS 3–8 score from EEG signals using family and nurse interaction experimental scenarios (tactile and auditory stimuli). Therefore, the present study aims to evaluate the GCS with objective data. The features were extracted from EEG signals in the time and frequency domains. After, the obtained features were statistically analyzed to differentiate the LeOC and to relate with GCS. The classification success of the proposed method for different LeOC was compared with various classifiers and evaluation metrics.

With our study, objective data were proposed for the unconscious patient group, which was difficult to evaluate using non-processed EEG signals. Differences between patients with consciousness levels 3 and 8 according to the GCS were revealed and classified using the proposed method. To the best of our knowledge, our study is the first in the literature in terms of the data recording stages, its purpose, and obtaining the results. So, our study is different and novel from other studies related to coma and EEG.

The main contributions of this study are as follows:

- The frequency analysis and machine learning methods used in this study may contribute to the detection of consciousness levels of patients in a deep coma and the development of BCI systems for objective determination of GCS.
- A new recording procedure including tactile and auditory stimuli has been proposed for this system, which was developed to examine changes in the EEG activity of different levels of consciousness and to measure the responses of patients to stimuli.
- Features extracted by power spectral density analysis from EEG signals could characterize the changes in brain function for a deep coma state. The results obtained may be valuable for future studies in predicting the prognosis of unconscious patients and are very important for further studies to show the difference in the levels of consciousness of patients in a deep coma.

This article is organized as follows. Section 2 describes the materials, including a detailed description of the proposed methods for the data collection, the pre-processing, and the features and analysis methods. Section 3 presents the results of features, statistical analysis, and machine learning algorithms. Section 4 presents a brief review of related works, discussions of the proposed method, and limitations of the study in classifying the LeOC. Section 5 is dedicated to the conclusion.

## 2. Materials and Methods

### 2.1. Subjects

The Erciyes University Hospital’s ICU patients were the subjects of our study. As the patients were unconscious and were unable to read and sign any document, all subjects’ families gave their informed consent before participating in this study.

The EEG signals analyzed in this study were recorded from 39 patients (19 males, 20 females, varying in age from 19 to 91 years, with a mean age of 67). Patients were in coma status (3 ≤ GCS ≤ 8). GCS of the patients was determined independently by two experienced clinicians. Inclusion criteria were (1) unconscious patients followed up in the ICU for any reason, (2) age ≥ 18 years, and (3) patients whose consciousness level was between 3 and 8 according to GCS. Exclusion criteria were (1) patients under sedation, (2) patients with suspected or realized brain death, (3) patients using any medication that would affect the brain waves, and (4) families that did not want to participate in the study. The Ethics Committee of Erciyes University Medical Faculty accepted the study protocol, and the research was carried out in compliance with the Helsinki Declaration. Table 1 summarizes the study population.

### 2.2. EEG Recordings and Pre-Processing

**Figure 2:**
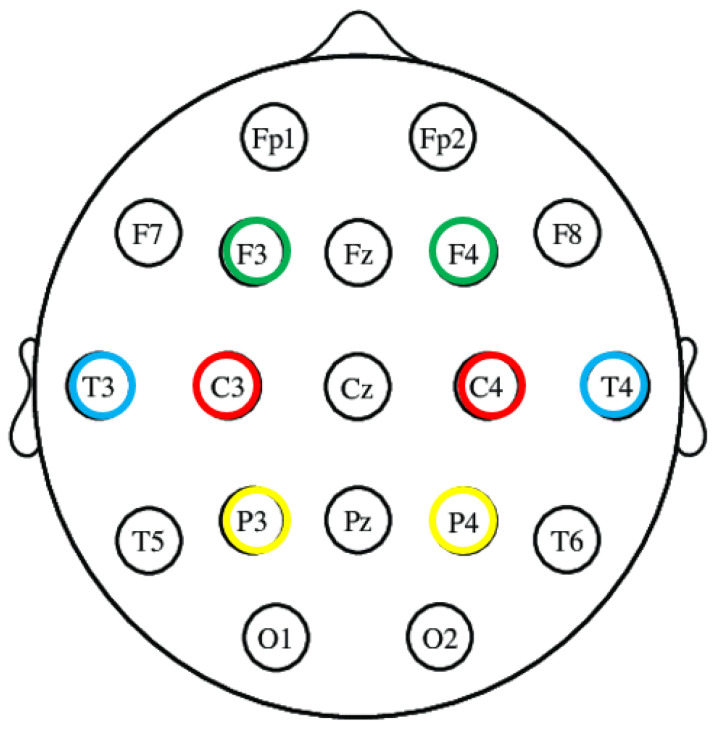
Electrode points of EEG recordings.

In this study, EEG signals were recorded continuously at a sampling frequency of 500 Hz using the standard 10–20 system of electrode placement. Data acquisition was performed using Biopac MP-150 System and EEG recordings were obtained using the EEG Cap and wet electrodes. During recordings, we used a bipolar montage with 4 bipolar channels (F3-F4/Channel 1, C3-C4/Channel 2, T3-T4/Channel 3, and P3-P4/Channel 4) (right earlobe is ground). The layout of eight electrodes is shown in Figure 2. During the recording, patients were lying down and at rest. A 50 Hz infinite impulse response notch filter and a finite impulse response lowpass filter with a cutoff frequency of 30 Hz were used to remove the artifacts before further analysis. Then, the denoised EEG signals were decomposed into six sub-bands: beta (13–30 Hz), alpha (8–13 Hz), theta (4–8 Hz), and delta (0.5–4 Hz).

**Figure 3:**
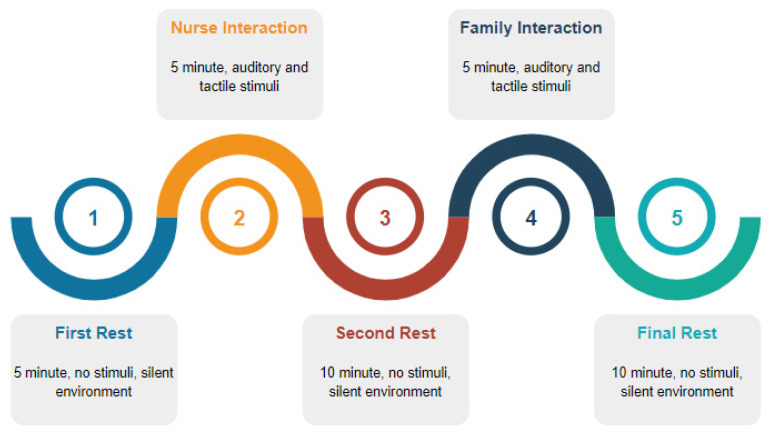
Proposed recording stages.

EEG signals were recorded from ICU patients in five stages, as seen in Figure 3, including three resting stages and nurse and family interaction (auditory, tactile stimuli) stages. During the first stage of the recording process, EEG signals were obtained in a silent environment without any stimulus for 5 min. Then, signal recordings were performed while the nurse who was routinely responsible for the patient spoke closely with the patient for 5 min. After the nurse interaction, resting stage EEG signals were recorded for 10 min. In the fourth stage of recordings, EEG signals were recorded while family members (patient relatives) interacted with the patient for 5 min. Finally, after interaction with the family as the last resting stage, EEG signals were recorded for 10 min. Thus, a total of 35 min of continuous EEG recording was obtained. During the patient’s interactions with the family and nurse, stimuli such as auditory stimuli (speaking with the patient) and tactile stimuli (touching the patient) were performed.

### 2.3. Power Spectral Density of EEG Signals and Feature Extraction

Spectrum estimation of signals is usually based on procedures operating a fast Fourier transform (FFT). The FFT approach has some limitations. Frequency resolution is one of these limitations. Another limitation is due to the windowing of the signal. Windowing exhibits itself as a ‘‘leakage’’ in the spectral domain. Moreover, FFT has high noise sensitivity and needs long-term data records for good frequency resolution [41]. Given the limitations of the FFT, Welch (non-parametric), Yule–Walker, and Burg AR (parametric) methods have been employed.

Power spectral density (PSD) is a crucial method in signal processing for frequency analysis. Parametric and non-parametric methods are used for PSD. Autoregressive, moving average, or autoregressive moving average use appropriate models with a known spectrum as parametric methods. Non-parametric methods do not have any assumption about the form of the power spectrum and estimate the PSD directly from the signal itself. Non-parametric methods are generally preferred because the estimated PSD may not be reliable if the model of the signal is not sufficiently and accurately defined using parametric methods [42,43,44]. In addition, there are studies indicating that parametric methods do not provide good performance for EEG signals [45].

The simplest non-parametric method is known as a periodogram. The periodogram method is based on Fourier and divides an EEG signal into frames of 64, 128, and 256, which are the powers of 2 [46]. The Welch method is a developed version of the periodogram and is used with an overlap of 50% in this study. This method (1) divides the signals into segments with a window function, computes a modified periodogram of each segment, and then averages the PSD estimates by (2) (*N*: length of the window, *L*: length of signal) [47].

(1) $$S\left(f\right)={\left|\frac{1}{N}\sum_{n=0}^{N-1}s\left(n\right)w\left(n\right)e^{-j2\pi fn}\right|}^{2}$$

(2) $$Pwelch\left(f\right)=\frac{1}{L}\sum_{i=1}^{L-1}S(f)$$

**Figure 4:**
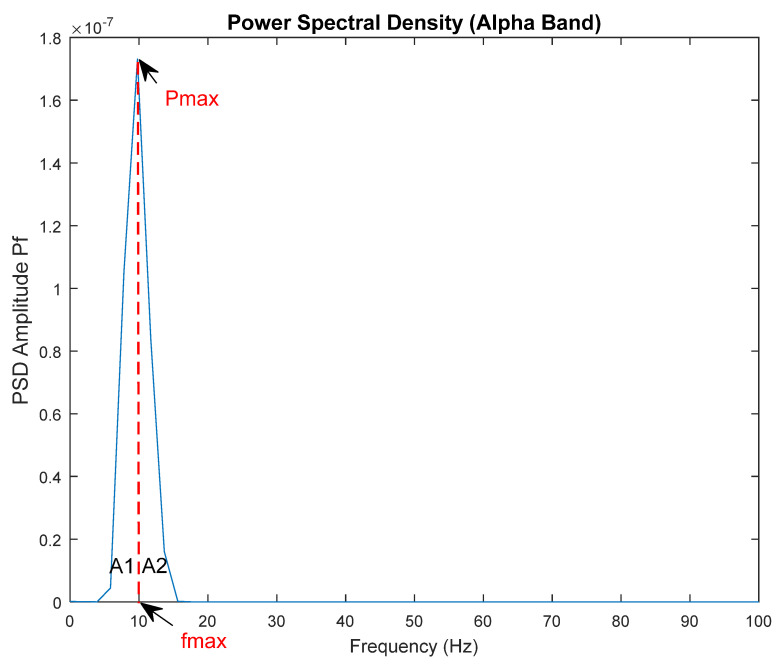
PSD graphic of F3-F4 channel EEG (a GCS 5 patient, first rest stage).

In this study, area and power values from the power spectrum graph, as seen in Figure 4, were calculated as features, and 10 features were extracted from each EEG sub-band and channel. Since the features of each EEG sub-band and channel are considered separate attributes, 160 features were created for an EEG signal. The energy of the EEG signal was calculated using the Parseval theorem (3) [48]. Features were calculated for all recording stages. A total of 10 min of resting EEG recordings were examined in 3 segments: the first 5 min, the last 5 min, and a total of 10 min of data. Since the recording stages were considered separate signals, the EEG signals belonging to a patient were divided into 9 segments: the 10 min rest signal (second rest and final rest), the initial five minutes, the last five minutes, and a total of ten minutes (a total of 6 segments), as well as nurse interaction signal, family interaction signal, and first rest signal. So, 345 instances were obtained because some rest stages’ signals could not be divided into 9 segments because they were not exactly 10 min. As a result, there were 160 features and 345 examples in this study. In Equations (4) and (5), *fs* is the sampling frequency and *Pf* is the power value of the signal. Areas for PSD curve of physiological signals obtained in patient control group distinction have recently come forth in the literature and gained importance. In the studies [46,47,48], the peak point of the curve was chosen as the reference point, and area calculations were made accordingly. Therefore, in this study, various features were extracted according to the area under the PSD curve [49,50,51]. The features are defined in Table 2.

(3) $$E_{x}^{frequency}=\frac{1}{N}\sum_{k=0}^{N-1}{\left|S(k)\right|}^{2}$$

(4) $$NPower=\frac{1}{fs}\sum_{k=1}^{N}{\left|Pf\right|}^{2}$$

(5) $$TPower=\sum_{k=1}^{N}{\left|Pf\right|}^{2}$$

### 2.4. Statistical Analysis

After the calculation of features from the EEG signal obtained at different stages, we performed the statistical analysis. As examined in the literature, most studies use the ANOVA test to compare multiple groups [49,50,51]. Therefore, we applied the normality test of Kolmogorov–Smirnov by considering *p* = 0.05 in the case of all statistical tests using the SPSS statistical software package. Normality assumptions were not satisfied for any one or both groups; therefore, the non-parametric test Kruskal–Wallis was applied. So, we used Kruskal–Wallis method to analyze the relationship between GCSs (GCS 3-8) and features obtained from EEG signals. Additionally, we used Dunn’s test to do pairwise comparisons between the features of the EEG signals in the case of different GCSs.

### 2.5. Data Balancing

Biomedical data classifications are very challenging because biomedical data is usually big and imbalanced. If the data distribution is highly unbalanced, classification algorithms may not perform correctly because they are intended to enhance overall accuracy with a bias toward the majority class, regardless of the relevance of the various classes [52]. The imbalance between the minority and majority classes of the data set misleads the classification results. For example, if 5% minority class instances are included in a data set, 95% majority class instances, most of the data, would be classified as majority class so that a very high accuracy rate can be achieved. However, the high accuracy rate does not indicate that the classification is correct. In such a case, minority data are misclassified [53].

Undersampling, oversampling, and hybrid method algorithms defined in three different groups can be mentioned to solve the unbalanced problem in the data [54]. In the literature, it is seen that oversampling methods are generally used. There are two types of oversampling methods: random and synthetic. The random oversampling method replicates instances from the minority class randomly. Even though random oversampling does not create new information since the instances are from existing entries, it increases the possibility of model overfitting. Therefore, synthetic oversampling methods have been developed to prevent overfitting. Synthetic oversampling methods produce artificial instances for the minority class [55]. In biomedical data sets, SMOTE is the most used oversampling method [56,57,58,59,60]. The SMOTE method was used in this study for the aim of data balancing.

The SMOTE method produces synthetic data based on the similarity of features by looking at the nearest neighbors (K neighbors) of the minority class samples (the nearest neighbors are randomly selected). In our study, we determined K = 5. The SMOTE technique uses the following formula to increase the number of instances of the minority class [61]. In this equation, *w_i_* is the coefficient of weight, and *x_knn_* is the sample for the K, the nearest neighbor of the minority class data.

(6) $$x_{new}=x_{i}+\left(x_{knn}-x_{i}\right)\times w_{i}$$

**Figure 5:**
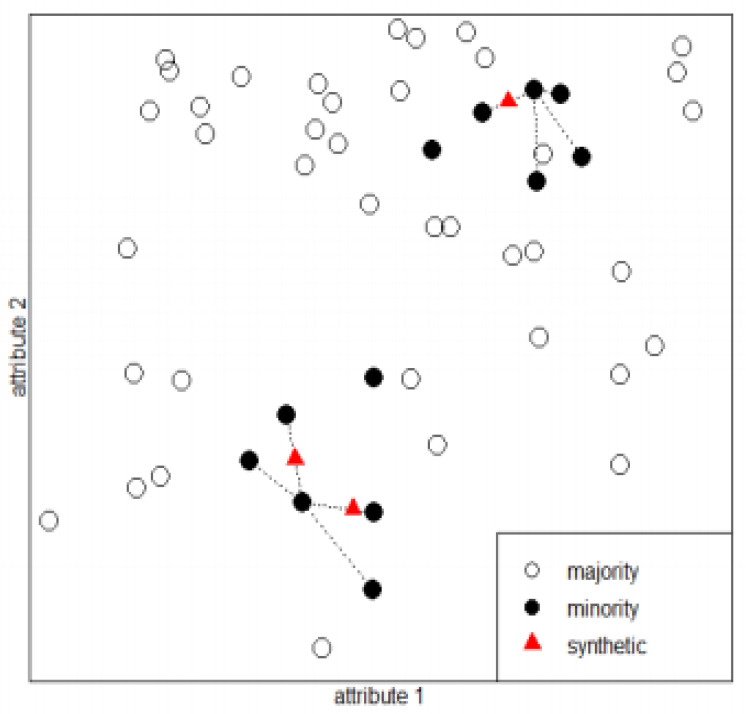
SMOTE application with K = 4.

Figure 5 shows the generation of synthetic data by looking at the nearest neighbor K = 4. White round specimens belong to the majority class and black round specimens belong to the minority class. As shown in Figure 5, *x_new_* synthetic data are generated by looking at 4 randomly selected samples around *x*_1_ and *x*_2_ samples.

### 2.6. Classification

Data Mining is extracting important patterns and discovering nontrivial knowledge from large data. Biomedical data mining is important in terms of finding the causes of the disease by examining the vital signs of a patient, providing the diagnosis of a disease with past data, and directing its treatment [62]. Classification is an important process in data mining and machine learning. A multiclass classification process was devised using the Weka and MATLAB software in this study. The usefulness of features obtained as a result of quantitative analysis of EEG for the diagnosis of neurological disorders using various classifiers has been discussed in many studies [63,64,65]. Random Forest (RF), K-NN, Ensemble Bagged Trees, and SVM-Cubic were used in this study. Grid search algorithm was used to find the optimal hyperparameters for each machine learning algorithm. For SVM, the kernel function was determined as cubic and the kernel scale as automatic. For the Ensemble Bagged Trees algorithm, the learner type was specified as the decision tree and the number of learners was determined as 30. For RF, the max iteration was specified as 100 with grid search in which we obtained the highest accuracy. For K-NN, the K value was determined as 1 and the distance metric was Euclidean. In all experiments, cross-validations were selected as values between 2 and 50 (k = 2, 5, 10, 15, 20, 25, 30, 35, 40, 50). Leave-one-out cross-validation procedure was also used to obtain the predicted labels for each patient in this study. GCS 3, GCS 4, GCS 5, GCS 6, GCS 7, and GCS 8 labels (for six classes) were applied for the classification of multiclass. In this study, only 20-fold cross-validation test results were included because the best accuracy rate was obtained for 20-fold.

The four algorithms whose classification performances were given in this study can be explained briefly as follows. SVM is a reliable approach for classifying nonlinear data. SVM creates a decision hyperplane to maximize the separation distance between various classes [66]. It then proceeds to create the accuracy value, which relies on the value of the kernel and the parameters that were used, after identifying the hyperplanes that separate the classes quite well.

RF is an ensemble algorithm in which features are selected randomly. A random sample of characteristics is chosen throughout the decision tree (DT) building process, and each tree independently forecasts a classification and “votes” for the associated class [67]. A single DT is noisier and more sensitive to outliers, and predictions are poorer than several DTs’ outputs. Thus, RF is desirable for medical data sets [68].

The K-NN algorithm is an algorithm that classifies the data to be classified according to its proximity with the previous data. The target point’s distance to be included in the sample data set from the existing data is calculated for the K number of neighbors and is allocated to the most common class among K nearest neighbors based on a majority vote of the neighbors [69].

The bagging method and the decision tree classifier are combined to form the Ensemble Bagged Trees classifier. The ensemble approach combines numerous machine learning classifiers, and the bagging method can minimize the decision tree algorithm’s high variance [70].

When working with unbalanced data sets, accuracy appears to be highly sensitive to data distribution resulting from the majority class. Misclassification of the minority class has a substantially greater error rate than the misclassification of the majority class. So, in addition to accuracy and error rate, other evaluation metrics should be used for imbalanced datasets [71]. The classifiers were evaluated in terms of measures for six class problems: sensitivity, specificity, precision, F-score, G-mean, and overall accuracy. Metrics were calculated according to Table 3. Accuracy is calculated as the ratio of correctly classified instances to the total number of instances (Equation (7)).

accuracy = (TP + TN)/(TP + TN + FP + FN)(7)

Sensitivity is also called Recall in some studies. Sensitivity measures a test’s capacity to appropriately detect true positive cases (those with disease). Specificity demonstrates the test’s capacity to appropriately detect real negative instances (those without the disease). Precision quantifies how many samples categorized as positive are actually positive [71].

sensitivity = TP/(TP + FN)(8)

specificity = TN/(TN + FP)(9)

precision = TP/(TP + FP)(10)

Sometimes, these metrics are not enough to indicate the success of classification studies. Therefore, in many studies, F-score and G-mean metrics were included [53]. The F-score is a classification metric that may be calculated as a weighted mean of accuracy and sensitivity. The *β* specified in the F-score equation is selected as one in a balanced state; i.e., precision and sensitivity are of equal weight. *β* is used to determine the significance of precision and sensitivity. In this study, *β* = 1 was determined [71].

(11) $$F-\mathrm{score}=\frac{\left(1+\beta^{2}\right)\times \mathrm{sensitivity}\times \mathrm{precision}}{\left(\beta^{2}\times \mathrm{sensitivity}\right)+\mathrm{precision}}$$

The G-mean (geometric mean) averages both sensitivity and specificity. Thus, it evaluates the degree of tendency in terms of both positive grade accuracy and negative grade accuracy rate. A low G-mean score, if interpreted, indicates that the classifier is prone to a class [71].

(12) $$G-\mathrm{mean}=\sqrt{\mathrm{sensitivity}\times \mathrm{specificity}}$$

## 3. Results

**Figure 6:**
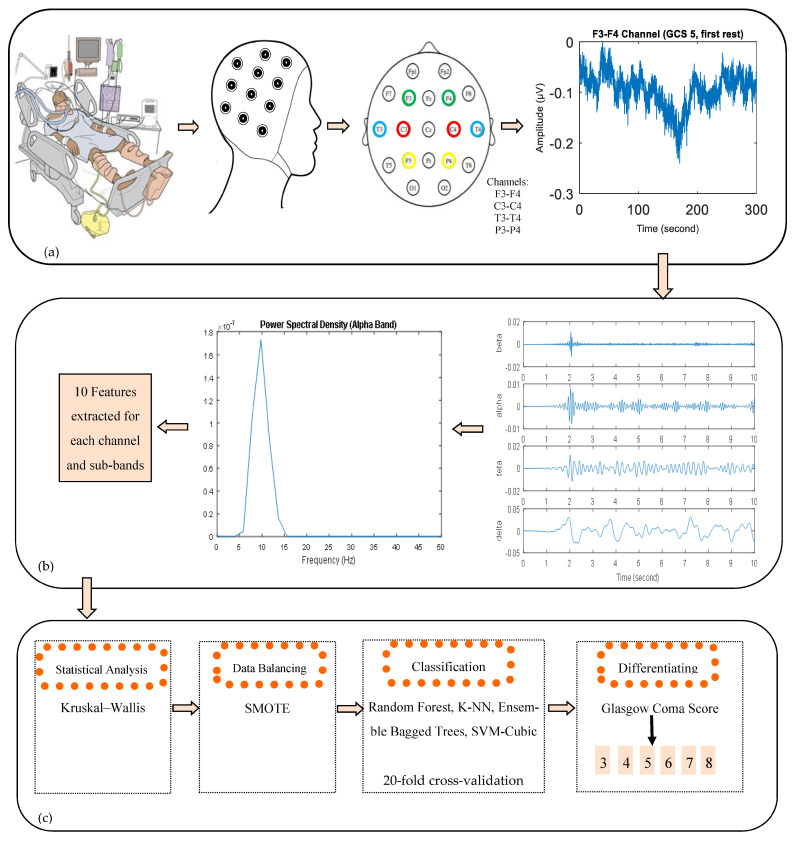
The main blocks of the presented methodology: (**a**) data acquisition and EEG recordings, (**b**) pre-processing and feature extraction, and (**c**) statistical analysis and classification.

In this study, 4 channels of EEG acquisition were performed from 39 comatose patients with GCSs between 3 and 8. The signals were recorded in five stages: first rest, interaction with a nurse, second rest, interaction with family, and last rest. During communication with the family and nurse, verbal and tactile stimuli were applied. The signals were filtered by a low-pass filter and notch filter and noises from signals were eliminated. Then, EEG signals were decomposed into four EEG sub-bands using a bandpass filter. The power spectrum density was obtained, and 10 features were extracted from the frequency spectrum for all sub-bands and each channel. Thus, 160 features in total were obtained. The features were statistically analyzed. The imbalance in the data was eliminated with the SMOTE method and classification results were given for 20-fold cross-validation. A schematic overview of the study is given in Figure 6.

### 3.1. Analysis of Energy Values

**Figure 7:**
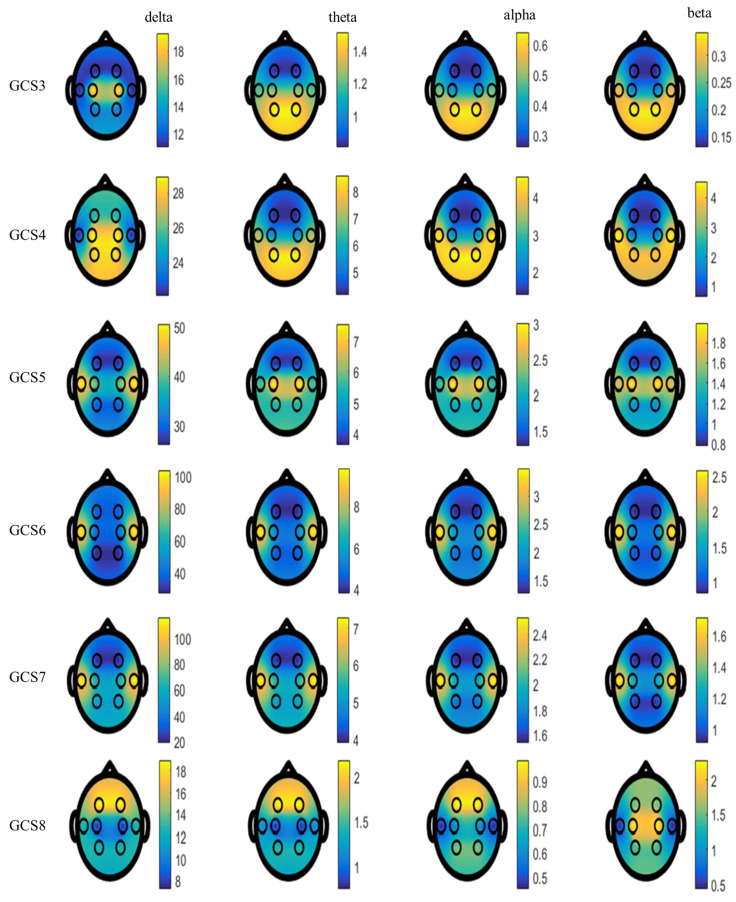
Topographical plots of mean energy values for sub-bands in GCS groups.

Energy values, which are the first feature in the study, were calculated for each EEG sub-band. The average ± standard error of means of energy values are shown in Table 4. In addition, topographic pictures of average energy values are given in Figure 7. According to Table 4, it is clearly seen that the energy values of GCS 3 patients in all sub-bands of each channel’s EEG signal are lower than other GCSs. The standard error mean of GCS 3 patients is lower than that of other GCSs. Therefore, it is understood that EEG signals of GCS 3 patients have less energy than other patients for all EEG channels and all sub-bands. As the patients’ GCS values increased, that is, their LeOC increased, their energy values also increased, as seen in Table 4. When we examine the delta band of the T3-T4 channel, it is seen that as the LeOC increases, the mean and standard deviation of energy values increase. The energy values of GCS 3 patients and GCS 8 patients were generally obtained close to each other, as seen in Figure 7. According to numerical results and topographical pictures, the EEG signals of GCS 3 patients have higher energy values in the parietal lobe in other sub-bands, except for the delta band. The parietal and temporal lobes of GCS 4 patients are more active. The central and temporal lobes of GCS 5 patients are active. The energy values of the EEG sub-bands of the temporal lobe are higher in patients with GCS 6 and GCS 7. In contrast, GCS 8 patients appear to have increased beta activity in the frontal lobe.

### 3.2. Statistical Analysis

It is important to analyze the features with statistical methods to understand the data. The conditions for parametric tests were examined. The Kruskal–Wallis method was used to statistically compare multiple groups since the data was not normally distributed. In this study, the extracted features were compared with different levels of consciousness. In other words, whether these features may distinguish patients with various GCS scores was explored. Most of the features were statistically significant between the groups (*p* < 0.05) according to the Kruskal–Wallis method. The results of the analysis are summarized in Table 5. In Table 5, the features that cannot make a statistical difference (*p* > 0.05) between groups are marked with red. It is seen that the EEG channel with the highest discrimination is T3-T4. Ratio 2 and Ratio 3 (seventh and eighth attributes) features were worse in distinguishing different levels of consciousness than other features. Post-hoc tests needed to be performed to find out which of the multiple groups differ from each other. For this, both groups were compared in pairs with the Dunn’s Bonferroni adjustment test. The results of which groups are different from each other are included in this study as a Supplementary Table S1.

**Figure 8:**
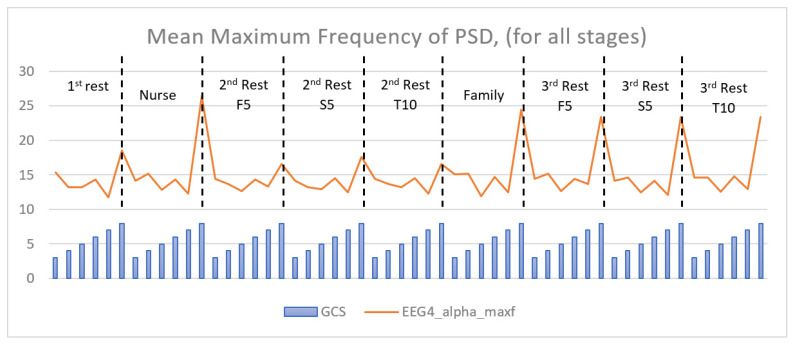
Mean values of the second feature (maxf) by different GCS groups. EEG4 represents the P3-P4 channel. Blue bars represent GCS 3, 4, 5, 6, 7, and 8, respectively, for each stage. F5: first five minutes, S5: second five minutes, T10: total ten minutes.

According to the results of comparing pairwise groups, it is the alpha sub-band of the P3-P4 channel that is best distinguished between multiple groups. For this reason, the average frequency values of the alpha band of the P3-P4 channel are plotted according to the GCS values. The mean frequency value of each recording stage according to the GCS is observed in Figure 8. If the graph is examined, it is seen that GCS 8 patients have the highest frequency in each recording stage and that GCS 6 shows a higher frequency content than other levels of consciousness. While the frequency features of GCS 3, 4, and 5 tend to decrease overall, those of GCS 6, 7, and 8 tend to increase.

**Figure 9:**
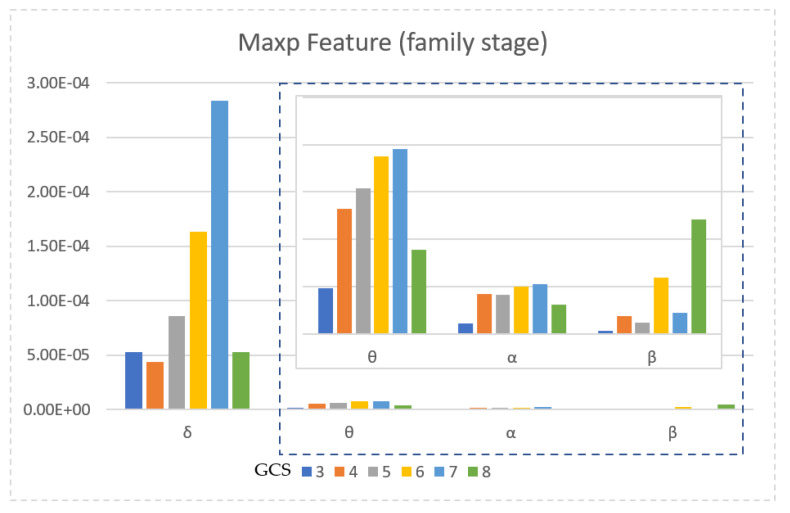
Mean of power values for all channels relative to sub-bands.

The power values according to the averaged sub-bands of all EEG channels are shown in Figure 9. Figure 9 shows the mean maximum power values of the EEG signals obtained during the interaction with the family because the power values of the family stage have been found to be more distinctive at different levels of consciousness. When Figure 9 is examined, it is seen that as the GCS value increases, the average power value of EEG signals increases. The EEG signals from GCS 3 patients have the lowest power value in each sub-band. Additionally, the EEG signals from GCS 8 patients have much a higher power in the beta sub-band than other levels of consciousness.

### 3.3. Data Balance

Since the number of GCS 8 patients are fewer than others, fewer instances were obtained from the EEG signals of these patients. If Table 6 is examined, the highest instance number is at GCS 6 with 81 instances, and the minimum instance number is at GCS 8 with 18 instances. If there are data with imbalanced instances between classes, greater success will be achieved in classifying the majority classes. For this reason, the SMOTE method has been used to overcome the multiclass imbalance problem. Instance numbers for different GCSs before and after using SMOTE are given in Table 6. Synthetic data were generated as much as the amount of increase (%) to approximately equal the number of instances. Thus, the imbalance problem between the classes was eliminated.

### 3.4. Classification Results

Classification results are shown in Table 7 using the classifiers described under the 2.6 classification subheading. The results are presented separately for the classification of unbalanced data and balanced data by SMOTE. The classification results are performed for analysis of all EEG sub-bands. The Random Forest algorithm achieves the best classification success rate. Furthermore, the fact that the data is balanced across many classes using the SMOTE approach improves classification success for all algorithms. An overall accuracy of 96.44% was obtained for the Random Forest algorithm. The second successful method was K-NN with a 96.23% overall accuracy, a classification performance very close to that of the Random Forest algorithm. The classification success is over 95% for the algorithms used in this study. When the classification success of the EEG sub-bands is measured, it can be seen from Table 8 that obtained features from the theta sub-band are more successful than the other EEG sub-bands in classifying different levels of consciousness.

**Figure 10:**
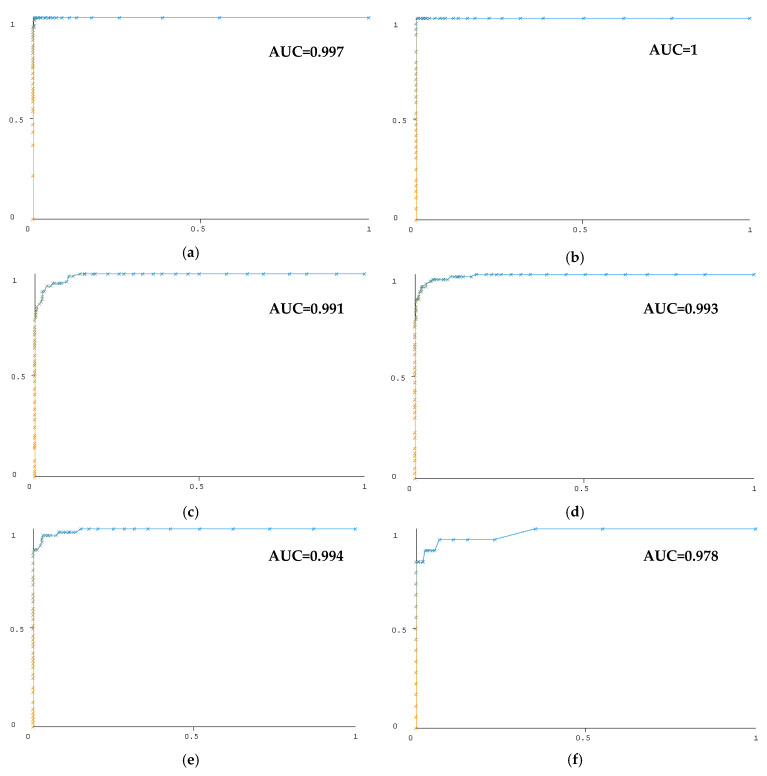
ROC curves for classification of *unbalanced* data with Random Forest. (**a**) GCS3 class, (**b**) GCS4 class, (**c**) GCS5 class, (**d**) GCS6 class, (**e**) GCS7 class, and (**f**) GCS8 class.

**Figure 11:**
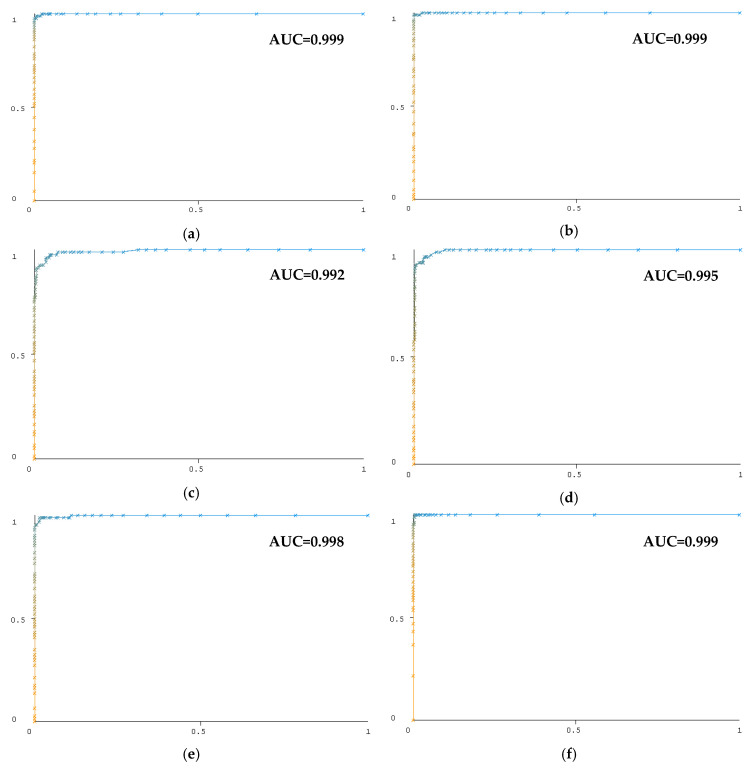
ROC curves for classification of balanced data with Random Forest. (**a**) GCS3 class, (**b**) GCS4 class, (**c**) GCS5 class, (**d**) GCS6 class, (**e**) GCS7 class, and (**f**) GCS8 class.

Figure 10 and Figure 11 show the ROC curves of the test data in the classification of six GCS classes from unbalanced and balanced data using the Random Forest algorithm. A separate curve is given for each GCS class. AUC stands for “Area under the ROC Curve”. The horizontal axis gives a false positive rate, and the vertical axis gives a true positive rate in the curves.

## 4. Discussion

When the energy values of EEG signals are analyzed, GCS 3 patients have lower energy in all sub-bands of all EEG channels. In the beta sub-band of the F3-F4 channel, an increased energy state is observed as GCS increases. The energies of EEG signals of GCS 4, GCS 5, and GCS 6 patients are close to each other. For GCS 8 patients, a higher energy value was obtained in the beta sub-band of EEG signals from only F3-F4 and C3-C4 channels than others. Through analysis of other channels and sub-bands, it is seen that the EEG signals of GCS 3 and GCS 8 patients have close energy values. The very low number of instances for GCS 8 patients causes low discrimination in other sub-bands except for the beta sub-band. If the topographic plots of the average energy values are examined, it is seen that the frontal lobe of GCS 8 patients is more active, and the parietal lobe of GCS 3 and GCS 4 patients is active. It was obtained that GCS 5, 6, and 7 patients had more activities in the temporal lobe. In the 10–20 system, the F7 point in EEG is near the centers for rational activities, the F8 point is near sources of emotional impulses, the C3 and C4 points deal with sensory and motor functions, the P3 and P4 promote the activity of perception and differentiation ability, and the T3 and T4 locations are concerned with emotional processors [72,73]. Since the frontal lobe is the brain region responsible for conscious thinking, we can conclude that GCS 8 patients have a higher LeOC. The parietal lobe integrates sensory input from numerous sections of the body and processes information related to touch [74]. The fact that the energy of the EEG channel of the parietal lobe of GCS 3 and GCS 4 patients is higher indicates that even the patients with very low awareness perceive the sense of touch. The temporal lobe plays a part in primary auditory perception, such as hearing [75]. Therefore, the temporal lobe of GCS 5, GCS 6, and GCS 7 patients being more active indicates that talking to these patients makes a difference in EEG waves.

When the statistical test results (Table 5) are examined, it is seen that most of the features reveal a substantial difference between the groups. As can be seen in Figure 8, during the interaction with the nurse and the family, talking to the patient/touching the patient causes the frequency content of the EEG signals of the patient (GCS 8 patients) to increase. It is also shown with the maximum power value that the change in the EEGs of GCS 8 patients is more clearly indicated. If the average power values for the sub-bands are examined in Figure 9, there can be seen an increasing power value as the LeOC increases. In this graph, which shows the power values of the family interaction stage, GCS 8 patients for the beta sub-band have a significantly higher power value than other levels of consciousness. The power values of the PSD graphics of GCS 3 patients are much lower than other levels of consciousness. As a result of the analysis of power values, GCS is correlated with the energy values.

Balancing the data with the SMOTE method for classifying consciousness levels has increased classification success. Figure 10 and Figure 11 show the influence of data balance on classification success, particularly in the classification of the GCS 8 class. The area under the ROC curve for balanced data is higher for the GCS 8 class than for unbalanced data. Classification with features obtained from the theta sub-band is performed with 93.10% success. Beta, alpha, and delta sub-bands have classifying successes close to each other. When a person is awake with their eyes closed, they produce alpha waves; when they are engaged in mental activity, they produce beta waves; theta waves occur between sleep and wakefulness; and delta waves occur during deep sleep [76]. Theta waves are usually related to drowsiness or heightened emotional states [77]. Theta frequency variations become more substantial during the shift from awake to sleepy states [78]. Therefore, the features extracted from theta waves have been more successful in classifying consciousness levels.

The statistical analysis of EEG shows a different pattern in frequency bands for different LeOC. During family and nurse interaction, a more complex organization of the EEG within the higher GCSs and changes in the distribution of the alpha and beta sub-band range were found. As a result, we were informed that patients between GCS 3 and 8 can be aware of what is going on around them, even when they are in a coma, by analyzing their EEG signals. Furthermore, our findings demonstrate that the energy, power, and frequency variations of EEG signals obtained from patients with different LeOCs can be used to objectively assess GCSs. Understanding that comatose patients are aware of their surroundings in ICUs will be beneficial for the patient’s care. This awareness can provide effective care and treatment services. Discontinuing useless care will result in the improved provision of psychological requirements in patient care.

### 4.1. Related Works

Currently, quantitative analysis studies show a distinctive change in EEG signals in the state of coma and brain death. Kustermann et al. [79] found that significant statistical differences in the spectral power of EEG obtained from comatose patients (within 24 h of cardiac arrest) occur at 5.2–13.2 Hz and above 21 Hz. According to [80], the power value indicated the great intension of neurophysiological EEG activities for the 19 coma patients, as well as the absence of these for 17 quasi-brain-death patients. Additionally, Zhu et al. [81] found that compared with the coma group, the relative power spectral density values of brain death were decreased in the delta band and increased in the alpha and beta bands. Claasen et al. [82] found a large decrease in power across all frequency bands. Lehembre et al. [83] compared differences in power spectra between VS patients and MCS patients. They noted that VS patients showed higher delta power but lower alpha power compared with MCS patients. Yao Miao et al. [84] indicated that the energy of EEG signals of comatose patients was higher than that of patients with brain death. Bai et al. [85] and Stefan et al. [86] found reduced power in the alpha range and increased power in the delta and theta range in VS patients compared with MCS patients. Finding differential diagnostics between MCS and VS is currently a challenge for researchers. Therefore, many studies are performed differentiating between VS and MCS (DOC patients) [87,88,89]. There is no study that has dealt with determining GCS levels in comatose patients (GCS 3-8) in the literature. However, there is a considerable amount of research on distinguishing DOC patients using EEG spectral power measures and other methodologies [32,33,87,88,89]. For example, Kempny et al. [90] compared the mean amplitudes of event-related potential data (ERP) in EEG recordings between VS/UWS and MCS patients. Statistical analysis was performed on the signals obtained by saying to the patients their own name and someone else’s name as auditory stimuli. Naro et al. [91] explored functional connectivity during resting-state EEG in 17 patients with VS/UWS and 15 patients with MCS using multiplex and multilayer network analyses. These studies vary from our study in that they do not aim to discriminate GCS scores in deep coma patients. The majority of the research is based on statistical analysis results. Our study contributes to the literature by demonstrating the usefulness of EEG spectral analysis in discriminating the LeOC in individuals in a deep coma.

When studies in the literature are examined, it is seen that studies are mostly aimed at differentiating between brain death and coma. There are not many studies to differentiate consciousness levels. The studies are mostly based on statistical analysis and classification studies are not encountered. This study is, to the best of our knowledge, the first in the literature in terms of the recording scenario, the extracted features, and the purpose. Some studies in the literature that analyze EEG signals with GCS are summarized in Table 9. These studies are different from our study and do not aim to separate the consciousness levels of patients in a deep coma. Therefore, our study contributes to the literature to represent the success of EEG signals in discriminating the LeOC.

In our prior research [97,98], we used deep neural networks to classify consciousness levels without extracting features from EEG recordings. As a result of this study, consciousness levels were classified into two classes (low level of consciousness and high level of consciousness) with 83.3% accuracy. In another study [98,99] we carried out, the classification of the level of consciousness with the features obtained as a result of the nonlinear analysis of the EEG signals was achieved with an accuracy of 90.3%. Unlike the previous two studies, the spectral content of the EEG waves was shown to provide discrimination in different levels of consciousness with an accuracy of 96.44% in this study.

### 4.2. Limitations and Feature Work

Although the proposed study achieved a high classification performance in different LeOC, there are still some limitations. First, the number of coma patients needs to be increased, especially the number of patients with GCS 8. Second, the difference between multiple groups for statistical analysis needs to be improved. Possible ways to improve statistical analysis are to ensure that the number of patients is equal for each GCS and to obtain new features. In future studies, we will overcome these limitations to better reveal the difference between levels of consciousness.

## 5. Conclusions

In this study, we proposed an EEG analysis system to differentiate different consciousness levels. For this purpose, EEG signals were recorded while the patients were applied with auditory and tactile stimuli by the nurse and their families; EEG signals were obtained before and after the stimuli. The features extracted from the sub-bands of EEG signals were statistically analyzed for different GCSs. While the features were successful in differentiating most GCS, this study demonstrates that GCS 3 and GCS 8 coma patients differ from other consciousness levels in terms of decreased theta sub-band energy values. This is, to the best of our knowledge, the first study to classify patients in a deep coma (GCS between 3 and 8) with 96.44% classification performance as a result of the recording and analysis method recommended. This study contributes to the literature with the results that EEG signals are successful in differentiating the LeOC in a deep coma.

## Figures and Tables

**Table 1:** Descriptive statistics of patients.

Glasgow Coma Scale	Number of Patients	Gender		Age	Time Since Onset (Days)
		Female	Male	Mean + SD	Mean + SD
3	8	4	4	71 ± 14	7 ± 4
4	4	2	2	76 ± 7	25 ± 18
5	9	6	3	74 ± 12	24 ± 19
6	9	5	4	64 ± 19	116 ± 140
7	7	3	4	62 ± 25	74 ± 108
8	2	-	2	34 ± 21	27 ± 21
Total	39	20	19	67 ± 19	51 ± 19

**Table 2:** List of 10 features extracted from each sub-band.

Feature	Explanation
Energy	The signal’s energy value; see Equation (3).
Maxf	Frequency value corresponding to the peak power in the PSD curve; see Figure 4.
Maxp	PSD’s maximum power value
AUC1	A1 area under the PSD curve, up to the peak power of PSD (as seen in Figure 4).
AUC2	A2 area of the curve after the PSD’s peak power (as seen in Figure 4).
Rate1	A1/(A1 + A2)
Rate2	A1/A2
Rate3	A2/(A1 + A2)
NPower	The entire power value, normalized *P_f_*; see Equation (4).
TPower	Total power; see Equation (5).

**Table 3:** Confusion matrix for GCS 3 class.

		Predicted Class					
		GCS3	GCS4	GCS5	GCS6	GCS7	GCS8
**Actual Class**	GCS3	TP (True Positive)	FN (False Negatives)	FN	FN	FN	FN
	GCS4	FP (False Positive)	TN (True Negatives)	TN	TN	TN	TN
	GCS5	FP	TN	TN	TN	TN	TN
	GCS6	FP	TN	TN	TN	TN	TN
	GCS7	FP	TN	TN	TN	TN	TN
	GCS8	FP	TN	TN	TN	TN	TN

**Table 4:** Energy values of different GCSs.

		Average Energy of Signal ± Standard Error of Mean					
	Channel	GCS3	GCS4	GCS5	GCS6	GCS7	GCS8
**Alpha**	F3-F4	0.2599 ± 0.0673	1.3997 ± 0.1812	1.2945 ± 0.1649	1.2653 ± 0.1623	1.5436 ± 0.4026	0.9883 ± 0.2727
	C3-C4	0.3754 ± 0.0497	2.3023 ± 0.3188	3.0098 ± 0.3541	2.0656 ± 0.2398	1.9960 ± 0.2122	0.5827 ± 0.1410
	T3-T4	0.4883 ± 0.0598	4.4359 ± 1.2724	2.1588 ± 0.3276	3.4919 ± 0.5847	2.5317 ± 0.4696	0.4524 ± 0.1500
	P3-P4	0.6420 ± 0.0829	4.5651 ± 1.0438	1.9746 ± 0.3957	1.6648 ± 0.1857	1.7292 ± 0.2369	0.7800 ± 0.2712
**Beta**	F3-F4	0.1297 ± 0.0318	0.6304 ± 0.0728	0.7827 ± 0.959	0.8539 ± 0.1751	0.9186 ± 0.2275	1.5938 ± 0.4470
	C3-C4	0.1760 ± 0.0213	1.4150 ± 0.2359	1.9921 ± 0.2959	1.3428 ± 0.2029	1.2878 ± 0.1959	2.2744 ± 0.8734
	T3-T4	0.3047 ± 0.0363	4.5623 ± 1.3450	1.7158 ± 0.2593	2.5749 ± 0.4866	1.7147 ± 0.3258	0.4410 ± 0.1489
	P3-P4	0.3432 ± 0.0446	4.0443 ± 1.0795	1.1305 ± 0.2628	0.9953 ± 0.1606	0.9440 ± 0.1509	1.4213 ± 0.3480
**Theta**	F3-F4	0.8103 ± 0.2217	4.1358 ± 0.5382	3.6714 ± 0.5268	3.8572 ± 0.4296	3.9233 ± 0.8821	2.1754 ± 0.7269
	C3-C4	1.2475 ± 0.2041	5.4085 ± 0.7598	7.5364 ± 0.8815	5.6074 ± 0.5775	5.5979 ± 0.6504	0.7547 ± 0.2462
	T3-T4	1.2003 ± 0.1863	7.2399 ± 2.0547	5.7090 ± 0.8948	9.7930 ± 1.4021	7.2743 ± 1.4515	1.1654 ± 0.3935
	P3-P4	1.5104 ± 0.1897	8.5885 ± 1.8550	5.4061 ± 1.0263	4.4837 ± 0.4578	5.0909 ± 0.6817	1.4382 ± 0.5709
**Delta**	F3-F4	11.0369 ± 3.0512	25.2814 ± 2.0644	26.6152 ± 3.9481	36.7705 ± 4.5119	19.4978 ± 2.6905	18.8845 ± 7.1786
	C3-C4	19.3139 ± 3.9593	28.9473 ± 2.8899	41.5905 ± 4.4525	43.1349 ± 6.9944	64.8723 ± 13.2537	7.2981 ± 2.5011
	T3-T4	11.1114 ± 1.6397	22.0236 ± 5.8738	50.7514 ± 6.1570	110.7027 ± 15.9699	116.4803 ± 32.1824	12.5969 ± 4.1904
	P3-P4	13.0764 ± 2.8072	28.3919 ± 5.6813	28.6629 ± 5.2250	27.5718 ± 3.0316	51.9683 ± 9.7944	12.6481 ± 4.1413

**Table 5:** Kruskal–Wallis test results.

	Channel 1 (F3-F4)				Channel 2 (C3-C4)				Channel 3 (T3-T4)				Channel 4 (P3-P4)			
Feature/ EEG Band	Alpha	Beta	Theta	Delta	Alpha	Beta	Theta	Delta	Alpha	Beta	Theta	Delta	Alpha	Beta	Theta	Delta
Energy	0.000	0.000	0.000	0.000	0.000	0.000	0.000	0.000	0.000	0.000	0.000	0.000	0.000	0.000	0.000	0.000
Maxf	0.000	0.000	0.002	0.056	0.083	0.000	0.054	0.163	0.000	0.000	0.001	0.009	0.000	0.000	0.000	0.055
Maxp	0.000	0.000	0.000	0.000	0.000	0.000	0.000	0.000	0.000	0.000	0.000	0.000	0.000	0.000	0.000	0.000
AUC1	0.000	0.000	0.000	0.000	0.000	0.000	0.000	0.000	0.000	0.000	0.000	0.000	0.000	0.000	0.000	0.000
AUC2	0.000	0.000	0.000	0.000	0.000	0.000	0.000	0.000	0.000	0.000	0.000	0.000	0.000	0.000	0.000	0.000
Rate1	0.000	0.000	0.000	0.000	0.000	0.000	0.000	0.000	0.000	0.000	0.000	0.000	0.000	0.000	0.000	0.000
Rate2	0.021	0.000	0.000	0.337	0.005	0.000	0.004	0.057	0.000	0.000	0.163	0.002	0.003	0.000	0.000	0.176
Rate3	0.021	0.000	0.000	0.336	0.005	0.000	0.004	0.057	0.000	0.000	0.163	0.002	0.003	0.000	0.000	0.176
NTPower	0.000	0.000	0.000	0.000	0.000	0.000	0.000	0.000	0.000	0.000	0.000	0.000	0.000	0.000	0.000	0.000
TPower	0.000	0.000	0.000	0.000	0.000	0.000	0.000	0.000	0.000	0.000	0.000	0.000	0.000	0.000	0.000	0.000

**Table 6:** Instance number by GCS.

GCS Score	Instance Number	Increase Amount (%)	Instance Number Using SMOTE
3	74	%8	79
*4*	36	%120	79
*5*	79	-	79
*6*	81	-	81
*7*	57	%40	81
*8*	18	%340	79
*Total*	345	%38.55	478

**Table 7:** Classification Results.

Imbalance Method	Classification Method	Sensitivity	Specificity	Precision	F-Score	G-Mean	Overall Accuracy
No	Random Forest	0.9343	0.9881	0.9605	0.9458	0.9604	0.9449
	K-NN	0.9049	0.9846	0.9439	0.9204	0.9426	0.9275
	Ensemble Bagged Trees	0.9137	0.9840	0.9397	0.9252	0.9478	0.9246
	SVM-Cubic	0.8664	0.9769	0.8972	0.8781	0.9183	0.8899
SMOTE	Random Forest	0.9644	0.9929	0.9653	0.9646	0.9785	0.9644
	K-NN	0.9625	0.9925	0.9624	0.9624	0.9773	0.9623
	Ensemble Bagged Trees	0.9561	0.9912	0.9563	0.9560	0.9734	0.9561
	SVM-Cubic	0.9293	0.9858	0.9289	0.9290	0.9568	0.9289

**Table 8:** Overall accuracy values are based on the features of different bands.

Data Balance	Algorithm	Alpha	Beta	Theta	Delta	All Band
Imbalance	RF	0.8870	0.8928	0.9159	0.8812	0.9449
Balance with SMOTE		0.9121	0.9121	0.9310	0.9247	0.9644

**Table 9:** Some of the studies aimed at differentiating coma levels.

Data	Subjects	Recording Time/Scenario	Methods and Features	Statistical Analysis	Classification	Study
EEG (19 channels)	20 coma patients	Not specified	Power spectral analysis and nonlinear analysis (Lempel-Ziv Complexity and entropy values)	Correlation *p* < 0.005	-	[21]
EEG (6 channels)	17 coma patients, 17 quasi-brain death patients	Not specified	Phase value with Shannon’s entopy	Independent sample *t*-test *p* < 0.0033	-	[25]
EEG (19 channels)	64 healthy subjects, 36 coma patients	Not specified	Lempel-Ziv complexity, approximate entropy, spectral entropy	*p* < 0.05 for two groups	-	[92]
EEG (6 channels)	2 coma patients	Recording at rest, (1535 s and 1030 s record)	Multivariate Empirical Mode Decomposition and Approximate Entropy	-	-	[93]
EEG (evoked potentials, ERP)	1 coma patient	Two experimental paradigms, word pairs and sentences	ANOVA, *t*-test, PCA-based t2 test, Wavelet	*p* ≤ 0.01	-	[94]
EEG (32 channels)	22 healthy subjects and 2 coma patients	Auditory odd-ball paradigm	Wavelet transform skewness, kurtosis, variance, maximum, minimum, and power values	-	Machine Learning Localized Feature Selection Method (%92.7 accuracy for healthy subjects)	[95]
EEG (5 channels) BT images	633 patients (GCS above 8)	10 min eyes closed resting	Fast Fourier transform, fractal analysis	-	Genetic Algorithm, Binary Classifier (96% sensitivity and 78% specificity for the structurally damaged group)	[96]
EEG (4 channels)	39 coma patiens (GCS ≤ 8)	35 min/tactile and auditory stimuli	Power spectral analysis	Kruskal–Wallis *p* < 0.000 in most features	Random Forest, SVM, Ensemble Bagged Trees, K-NN 96.44% accuracy	Proposed study

## Data Availability

The data presented in this study are available on request from the corresponding author.

## Supplementary Materials

The following supporting information can be downloaded at https://www.mdpi.com/article/10.3390/diagnostics13081383/s1: Table S1: Kruskal–Wallis test post-hoc results (Dunn’s test, Bonferroni correction).

## References

[1] Cooksley T., Holland M. (2013). The unconscious patient. Medicine.

[2] Campbell S., McCormick W. (2002). Approach to the comatose patient. Can. J. CME.

[3] Rapsang A.G., Shyam D.C. (2014). Scoring systems in the intensive care unit: A compendium. Indian J. Crit. Care Med..

[4] Karabıyık L. (2010). Yoğun Bakımda Skorlama Sistemleri. Yoğun Bakım Derg..

[5] Sakarya M. (2006). Skorlama Sistemleri. Türk Yoğun Bakım Derneği Derg..

[6] Rosenfeld J.V., Lennarson P.J., Schapira A.H.V., Byrne E., Frackowiak R.S.J., Mizuno Y., Silberstein S.D. (2007). Coma and Brain Death. Neurology and Clinical Neuroscience.

[7] Zheng W.B., Liu G.R., Kong K.M., Wu R.H. Coma Duration Prediction in Diffuse Axonal Injury: Analyses of Apparent Diffusion Coefficient and Clinical Prognostic Factors. Proceedings of the 28th Annual International Conference of the IEEE Engineering in Medicine and Biology Society.

[8] Teasdale G., Jennett B. (1974). Assessment of coma and impaired consciousness. A practical scale. Lancet.

[9] Namiki J., Yamazaki M., Funabiki T., Hori S. (2011). Inaccuracy and misjudged factors of Glasgow Coma Scale scores when assessed by inexperienced physicians. Clin. Neurol. Neurosurg..

[10] (2000). Brain Trauma Foundation and American Association of Neurological Surgeons, Early indicators of prognosis in severe traumatic brain injury, Glasgow Coma Scale score. J. Neurotrauma.

[11] Crossman J., Bankes M., Bhan A., Crockard H.A. (1998). The Glasgow Coma Score: Reliable evidence?. Injury.

[12] Gill M.R., Reiley D.G., Green S.M. (2004). Interrater reliability of Glasgow Coma Scale scores in the emergency department. Ann. Emerg. Med..

[13] Riechers R.G., Ramage A., Brown W., Kalehua A., Rhee P., Ecklund J.M., Ling G.S., Reith F.C., Brennan P.M., Maas A.I. (2005). Physician Knowledge of the Glasgow Coma Scale. J. Neurotrauma.

[14] Rowley G., Fielding K. (1991). Reliability and accuracy of the Glasgow Coma Scale with experienced and inexperienced users. Lancet.

[15] Schnakers C., Vanhaudenhuyse A., Giacino J., Ventura M., Boly M., Majerus S., Moonen G., Laureys S. (2009). Diagnostic accuracy of the vegetative and minimally conscious state: Clinical consensus versus standardized neurobehavioral assessment. BMC Neurol..

[16] Wieser M., Koenig B.A., Riener R. Quantitative Description of the State of Awareness of Patients in Vegetative and Minimally Conscious State. Proceedings of the 32nd Annual International Conference of the IEEE EMBS.

[17] Goldberg S.A., Rojanasarntikul D., Jagoda A., Grafman J., Andres Salazar M. (2015). The prehospital management of traumatic brain injury. Handbook of Clinical Neurology.

[18] Laureys S. (2005). The neural correlate of (un)awareness: Lessons from the vegetative state. Trends Cogn. Sci..

[19] Tarassenko L., Clifton D., Pinksky M., Hravnak M., Woods J., Watkinson P. (2011). Centile-based early warning scores derived from Statistical distributions of vital signals. Resuscitation.

[20] Tarassenko L., Hann A., Young D. (2006). Integrated monitoring and analysis for early warning of patient deterioration. Brit. J. Anaesthesia.

[21] Lin M.A., Chan H.L., Fang S.C. Linear and Nonlinear EEG Indexes in Relation to the Severity of Coma. Proceedings of the 27th Annual International Conference of the IEEE Engineering in Medicine and Biology Society.

[22] Van Gils M., Rosenfalck A., White S., Prior P., Gade J., Senhadji L., Thomsen C., Ghosh I., Longford R., Jensen K. (1997). Signal processing in prolonged EEG recordings during intensive care. IEEE Eng. Med. Biol. Mag..

[23] Shah A.K., Agarwal R., Carhuapoma J., Loeb J.A. (2006). Compressed EEG Pattern Analysis for Critically Ill Neurological-Neurosurgical Patients. Neurocrit. Care.

[24] Flower L. (2016). Literature Survey on Biomedical Signal Processing Methods. Int. J. Innov. Res. Comput. Commun. Eng..

[25] Li L., Witon A., Marcora S., Bowman H., Mandic D.P. EEG-Based Brain Connectivity Analysis of States of Unawareness. Proceedings of the 36th Annual International Conference of the IEEE Engineering in Medicine and Biology Society.

[26] Ahmed B., Tafreshi R., Langari R. The Future of Automatic EEG Monitoring in the Intensive Care. Proceedings of the International Conference on BioMedical Engineering and Informatics.

[27] Hussain I., Park S.J. (2020). HealthSOS: Real-Time Health Monitoring System for Stroke Prognostics. IEEE Access.

[28] Hussain I., Park S.-J. (2021). Quantitative Evaluation of Task-Induced Neurological Outcome after Stroke. Brain Sci..

[29] Islam M.S., Hussain I., Rahman M., Park S.J., Hossain A. (2022). Explainable Artificial Intelligence Model for Stroke Prediction Using EEG Signal. Sensors.

[30] Kotchoubey B. (2017). Evoked and event-related potentials in disorders of consciousness: A quantitative review. Conscious. Cogn..

[31] Mikola A., Särkelä M.O., Walsh T.S., Lipping T. Power Spectrum and Cross Power Spectral Density Based EEG Correlates of Intensive Care Delirium. Proceedings of the 41st Annual International Conference of the IEEE Engineering in Medicine and Biology Society (EMBC).

[32] Lechinger J., Bothe K., Pichler G., Michitsch G., Donis J., Klimesch W., Schabus M. (2013). CRS-R score in disorders of consciousness is strongly related to spectral EEG at rest. J. Neurol..

[33] Piarulli A., Bergamasco M., Thibaut A., Cologan V., Gosseries O., Laureys S. (2016). EEG ultradian rhythmicity differences in disorders of consciousness during wakefulness. J. Neurol..

[34] Naro A., Bramanti P., Leo A., Cacciola A., Bramanti A., Manuli A., Calabrò R.S. (2016). Towards a method to differentiate chronic disorder of consciousness patients’ awareness: The Low-Resolution Brain Electromagnetic Tomography Analysis. J. Neurol. Sci..

[35] Kotchoubey B., Lang S., Mezger G., Schmalohr D., Schneck M., Semmler A., Bostanov V., Birbaumer N. (2005). Information processing in severe disorders of consciousness: Vegetative state and minimally conscious state. Clin. Neurophysiol..

[36] Khanmohammadi S., Laurido-Soto O., Eisenman L.N., Kummer T.T., Ching S. (2018). Intrinsic network reactivity differentiates levels of consciousness in comatose patients. Clin. Neurophysiol..

[37] Serafmi G., Acra W., Scuteri F., Palmieri A.M.R., Simoncelli C., Serafini G. (1994). Auditory Evoked Potentials at 40 Hz (SSR40Hz) in Post-Trauma Coma Patients. Laryngoscope.

[38] Firsching R., Luther J., Eidelberg E., Brown W.E., Story J.L., Boop F.A. (1987). 40 Hz—middle latency auditory evoked response in comatose patients. Electroencephalogr. Clin. Neurophysiol..

[39] Binder M., Górska U., Griskova-Bulanova I. (2017). 40 Hz auditory steady-state responses in patients with disorders of consciousness: Correlation between phase-locking index and Coma Recovery Scale-Revised score. Clin. Neurophysiol..

[40] Chen T., Lu S., Qian P., Chen G., Hu N. (2020). An automatic detection method for 40-Hz auditory steady state response and its application in prognosis of comatose patients. Clin. Neurophysiol..

[41] Kailath T. (1986). Modern Signal Processing.

[42] Proakis J.G., Manolakis D.G. (2007). Digital Signal Processing: Principles, Algorithms, and Applications.

[43] Ariananda D.D., Lakshmanan M.K., Nikookar H. A survey on spectrum sensing techniques for cognitive radio. Proceedings of the 2009 Second International Workshop on Cognitive Radio and Advanced Spectrum Management.

[44] Zhao H., Gui L. (2019). Nonparametric and parametric methods of spectral analysis. MATEC Web Conf..

[45] Shiman F., Safavi S.H., Vaneghi F.M., Oladazimi M., Safari M.J., Ibrahim F. EEG feature extraction using parametric and non-parametric models. Proceedings of the 2012 IEEE-EMBS International Conference on Biomedical and Health Informatics.

[46] Welch P.D. (1967). The use of fast Fourier transform for the estimation ofpower spectra: A method based on time averaging over short, modified periodograms. IEEE Trans. Audio Electroacoust..

[47] Stoica P., Moses R. (2005). Spectral Analysis of Signals.

[48] Chaparro L. (2015). Frequency Analysis: The Fourier Series. Signals and Systems Using MATLAB.

[49] Kara S., Latifoğlu F. Analysis of internal carotid artery and ophthalmic artery Doppler signals using discrete wavelet transformation. Proceedings of the European Symposium on Biomedical Engineering.

[50] Batbat T., Güven A., Dolu N. (2019). Evaluation of divided attention using different stimulation models in event-related potentials. Med Biol. Eng. Comput..

[51] Güven A., Altınkaynak M., Dolu N., Ünlühızarcı K. (2015). Advanced analysis of auditory evoked potentials in hyperthyroid patients: The effect of filtering. J. Med. Syst..

[52] Anand A., Pugalenthi G., Fogel G.B., Suganthan P.N. (2010). An approach for classification of highly imbalanced data using weighting and undersampling. Amino Acids.

[53] Yan Y., Liu R., Ding Z., Du X., Chen J., Zhang Y. (2019). A Parameter-Free Cleaning Method for SMOTE in Imbalanced Classification. IEEE Access.

[54] Esteves V.M.S. (2020). Techniques to Deal with Imbalanced Data in Multi-Class Problems: A Review of Existing Methods. Master’s Thesis.

[55] Shelke M.S., Deshmukh P.R., Shandilya V.K. (2017). A Review on Imbalanced Data Handling Using Undersampling and Oversampling Technique. Int. J. Recent Trends Eng. Res..

[56] Wang Y., Simon M., Bonde P., Harris B.U., Teuteberg J.J., Kormos R.L., Antaki J.F. (2012). Prognosis of Right Ventricular Failure in Patients With Left Ventricular Assist Device Based on Decision Tree With SMOTE. IEEE Trans. Inf. Technol. Biomed..

[57] Lijun L., Tingting L., Meiya H. Feature identification from imbalanced data sets for diagnosis of Cardiac Arrhythmia. Proceedings of the 11th International Symposium on Computational Intelligence and Design (ISCID).

[58] Shree B., Sheshadri B.S. An approach to preprocess data in the diagnosis of Alzheimer’s Disease. Proceedings of the 2014 International Conference on Cloud Computing and Internet of Things.

[59] Gao R., Peng J., Nguyen L., Liang Y., Thng S., Lin Z. Classification of Non-Tumorous Facial Pigmentation Disorders using Deep Learning and SMOTE. Proceedings of the IEEE International Symposium on Circuits and Systems (ISCAS).

[60] Abdoh S.F., Rizka M.A., Maghraby F.A. (2018). Cervical Cancer Diagnosis Using Random Forest Classifier With SMOTE and Feature Reduction Techniques. IEEE Access.

[61] Chawla N.V., Bowyer K.W., Hall L.O., Kegelmeyer W.P. (2002). SMOTE: Synthetic Minority Over-sampling Technique. J. Artif. Intell. Res..

[62] Bou Rjeily C., Badr G., Hajjarm El Hassani A., Andres E., Tsihrintzis G., Sotiropoulos D., Jain L. (2018). Medical Data Mining for Heart Diseases and the Future of Sequential Mining in Medical Field. Machine Learning Paradigms. Intelligent Systems Reference Library.

[63] Prichep L., John E., Ferris S., Rausch L., Fang Z., Cancro R., Torossian C., Reisberg B. (2006). Prediction of longitudinal cognitive decline in normal elderly with subjective complaints using electrophysiological imaging. Neurobiol. Aging.

[64] Thatcher R.W., North D.M., Curtin R.T., Walker R.A., Biver C.J., Gomez J.F., Salazar A.M. (2001). An EEG severity index of traumatic brain injury. J. Neuropsychiatry Clin. Neurosci..

[65] Cao C., Tutwiler R.L., Slobounov S. (2008). Automatic Classification of Athletes With Residual Functional Deficits Following Concussion by Means of EEG Signal Using Support Vector Machine. IEEE Trans. Neural Syst. Rehabil. Eng..

[66] Cortes C., Vapnik V. (1995). Support-vector networks. Mach. Learn..

[67] Breiman L. (2001). Random forests. Mach. Learn..

[68] Podgorelec V., Kokol P., Stiglic B., Rozman I. (2002). Decision trees: An overview andtheir use in medicine. J. Med. Syst..

[69] Gou J., Ma H., Ou W., Zeng S., Rao Y., Yang H. (2019). A generalized mean distance-based k-nearest neighbor classifier. Expert Syst. Appl..

[70] Zhou Z.H. (2012). Ensemble Methods: Foundations and Algorithms. Bookshelf.

[71] Gosain A., Sardana S. Handling Class Imbalance Problem using Oversampling Techniques: A Review. Proceedings of the 2017 ICACCI.

[72] Michal T. (2002). Fundamental of EEG Measurement. Meas. Sci. Rev..

[73] Nunez P.L. (1995). Neocortical Dynamics and Human EEG Rhythms.

[74] Blakemore S.-J., Frith U. (2005). The Learning Brain.

[75] Schacter D.L., Gilbert D.T., Wegner D.M. (2010). Psychology.

[76] Niedermeyer E., da Silva F.L. (2005). Electroencephalography: Basic Principles, Clinical Applications, and Related Fields.

[77] Scher M. (2017). Pediatric Neurophysiologic Evaluation, Swaiman’s Pediatric Neurology.

[78] Mari-Acevedo J., Yelvington K., Tatum W.O. (2019). Normal EEG variants. Handbook of Clinical Neurology.

[79] Kustermann T., Nguissi N.A.N., Pfeiffer C., Haenggi M., Kurmann R., Zubler F., Oddo M., Rossetti A.O., De Lucia M. (2019). Electroencephalography-based power spectra allow coma outcome prediction within 24 h of cardiac arrest. Resuscitation.

[80] Shi Q., Yang J., Cao J., Tanaka T., Wang R., Zhu H. (2011). EEG data analysis based on EMD for coma and quasi-brain-death patients. J. Exp. Theor. Artif. Intell..

[81] Zhu L., Cui G., Cao J., Cichocki A., Zhang J., Zhou C. (2019). A Hybrid System for Distinguishing between Brain Death and Coma Using Diverse EEG Features. Sensors.

[82] Claassen J., Velazquez A., Meyers E., Witsch J., Falo M.C., Park S., Agarwal S., Schmidt J.M., Schiff N.D., Sitt J.D. (2016). Bedside quantitative electroencephalography improves assessment of consciousness in comatose subarachnoid hemorrhage patients. Ann. Neurol..

[83] Lehembre R., Marie-Aurélie B., Vanhaudenhuyse A., Chatelle C., Cologan V., Leclercq Y., Soddu A., Macq B., Laureys S., Noirhomme Q. (2012). Resting-state EEG study of comatose patients: A connectivity and frequency analysis to find differences between vegetative and minimally conscious states. Funct. Neurol..

[84] Miao Y., Cao J. (2018). Descriptive statistical analysis based on patients EEG energy in coma and quasi-brain-death state. Int. J. Comput. Technol..

[85] Bai Y., Xia X., Li X., Wang Y., Yang Y., Liu Y., Liang Z., He J. (2017). Spinal cord stimulation modulates frontal delta and gamma in patients of minimally consciousness state. Neuroscience.

[86] Stefan S., Schorr B., Lopez-Rolon A., Kolassa I.-T., Shock J.P., Rosenfelder M., Heck S., Bender A. (2018). Consciousness Indexing and Outcome Prediction with Resting-State EEG in Severe Disorders of Consciousness. Brain Topogr..

[87] Bagnato S., Boccagni C., Sant’Angelo A., Prestandrea C., Mazzilli R., Galardi G. (2015). EEG predictors of outcome in patients with disorders of consciousness admitted for intensive rehabilitation. Clin. Neurophysiol..

[88] Estraneo A., Loreto V., Guarino I., Boemia V., Paone G., Moretta P., Trojano L. (2016). Standard EEG in diagnostic process of prolonged disorders of consciousness. Clin. Neurophysiol..

[89] Corchs S., Chioma G., Dondi R., Gasparini F., Manzoni S., Markowska-Kacznar U., Mauri G., Zoppis I., Morreale A. (2019). Computational Methods for Resting-State EEG of Patients With Disorders of Consciousness. Front. Neurosci..

[90] Kempny A.M., James L., Yelden K., Duport S., Farmer S., Playford E.D., Leff A. (2018). Patients with a severe prolonged Disorder of Consciousness can show classical EEG responses to their own name compared with others’ names. NeuroImage Clin..

[91] Naro A., Maggio M.G., Leo A., Calabrò R.S. (2021). Multiplex and Multilayer Network EEG Analyses: A Novel Strategy in the Differential Diagnosis of Patients with Chronic Disorders of Consciousness. Int. J. Neural Syst..

[92] Chan H.L., Lin M.A., Fang S.C. Linear and Nonlinear Analysis of Electroencephalogram of the Coma. Proceedings of the 26th Annual International Conference of the IEEE EMBS.

[93] Cui G., Yin Y., Zhao Q., Cichocki A., Cao J. Patients’ consciousness analysis using Dynamic Approximate Entropy and MEMD method. Proceedings of the 2013 Asia-Pacific Signal and Information Processing Association Annual Summit and Conference.

[94] Kotchoubey B., Daltrozzo J., Wioland N., Mutschler V., Lutun P., Birbaumer N., Jaeger A. (2005). Semantic processing in a coma patient. Grand Rounds.

[95] Armanfard N., Komeili M., Reilly J.P., Connolly J.F. (2019). A Machine Learning Framework for Automatic and Continuous MMN Detection With Preliminary Results for Coma Outcome Prediction. IEEE J. Biomed. Health Inform..

[96] Prichep L.S., Jacquin A., Filipenko J., Dastidar S.G., Zabele S., Vodencarevic A., Rothman N.S. (2012). Classification of Traumatic Brain Injury Severity Using Informed Data Reduction in a Series of Binary Classifier Algorithms. IEEE Trans. Neural Syst. Rehabil. Eng..

[97] Altıntop G., Latifoğlu F., Akın A.K., Çetin B. (2022). A novel approach for detection of consciousness level in comatose patients from EEG signals with 1-D convolutional neural network. Biocybern. Biomed. Eng..

[98] Altıntop Ç.G. (2021). Determination of Consciousness Levels by Analysis and Classification of Physiological Signals of Deep Coma Patients in Intensive Care Units. Ph.D. Thesis.

[99] Altıntop Ç.G., Latifoğlu F., Akın A.K., Bayram A., Çiftçi M. (2022). Classification of Depth of Coma Using Complexity Measures and Nonlinear Features of Electroencephalogram Signals. Int. J. Neural Syst..

