# Supplementary material for: Quantitative Electroencephalography Analysis for Improved Assessment of Consciousness Levels in Deep Coma Patients Using a Proposed Stimulus Stage
Source: Diagnostics (Basel). 2023 Apr 10;13(8):1383. doi: 10.3390/diagnostics13081383 (PMC10137534; doi:10.3390/diagnostics13081383)
Supplement: Supplementary file 1 [file diagnostics-13-01383-s001.zip › diagnostics-2290547-supplementary.pdf]

diagnostics

MDPI

Supplementary Material: Quantitative Electroencephalography Analysis for Improved Assessment of Different Consciousness Level in Comatose Patients Using a Proposed Stimulus Stage

Çiğdem Gülüzar Altintop, Fatma Latifoğlu, Aynur Karayol Akin, and Ayşe Ülgey

Table S1. Kruskal Wallis test post-hoc results (Dunn's test, Bonferroni correction).

Feature/ EEG Band	Channel 1 (F3-F4)				Channel 2 (C3-C4)				Channel 3 (T3-T4)				Channel 4 (P3-P4)			
	alpha	beta	theta	delta	alpha	beta	theta	delta	alpha	beta	theta	delta	alpha	beta	theta	delta
1st Feature Energy	3-4<br>3-5<br>3-6<br>3-7<br>3-8	3-4<br>3-5<br>3-6<br>3-7<br>3-8	3-4<br>3-5<br>3-6<br>3-7<br>3-8	3-4<br>3-5<br>3-6<br>3-7	3-4<br>3-5<br>3-6<br>3-7<br>4-8<br>5-8<br>7-8	3-4<br>3-5<br>3-6<br>3-7<br>3-8	3-4<br>3-5<br>3-6<br>3-7<br>4-8<br>5-8<br>6-8<br>7-8	3-4<br>3-5<br>3-6<br>3-7<br>4-8<br>5-8<br>6-8<br>7-8	3-5<br>3-6<br>3-7<br>5-8<br>6-8	3-4<br>3-5<br>3-6<br>3-7<br>4-6<br>6-7<br>6-8	3-5<br>3-6<br>3-7<br>4-6<br>5-8<br>6-8	3-5<br>3-6<br>3-7<br>4-5<br>4-6<br>5-8<br>6-7<br>6-8	3-4<br>3-6<br>3-7<br>4-8	3-4<br>3-5<br>3-6<br>3-7<br>5-8	3-4<br>3-5<br>3-6<br>3-7<br>4-8<br>6-8<br>7-8	3-4<br>3-6<br>3-7<br>5-7<br>7-8
2nd Feature fmax	3-4<br>3-5<br>3-6<br>3-7	3-6<br>3-8<br>4-8<br>5-8<br>6-8<br>7-8	3-6<br>6-7	**	**	3-8<br>4-8<br>5-8<br>6-8<br>7-8	**	**	3-5<br>4-5	3-8<br>4-8<br>5-8<br>6-8<br>7-8	3-6<br>4-6	3-6<br>3-7	3-5<br>3-7<br>4-5<br>4-7<br>5-8<br>6-7<br>6-5<br>7-8	3-6<br>3-8<br>4-8<br>5-8<br>6-8<br>7-8	3-4<br>4-6<br>4-7<br>5-7	**
3rd Feature Pmax	3-4<br>3-5<br>3-6<br>3-7<br>3-8	3-4<br>3-5<br>3-6<br>3-7<br>3-8	3-4<br>3-5<br>3-6<br>3-7	3-4<br>3-5<br>3-6<br>3-7<br>6-8	3-4<br>3-5<br>3-6<br>3-7<br>5-8	3-4<br>3-5<br>3-6<br>3-7<br>3-8	3-4<br>3-5<br>3-6<br>3-7<br>4-8<br>5-8<br>6-8<br>7-8	3-4<br>3-5<br>3-6<br>3-7<br>4-8<br>5-8<br>6-8<br>7-8	3-5<br>3-6<br>3-7<br>4-6<br>4-8<br>5-8<br>6-7<br>6-8<br>7-8	3-5<br>3-6<br>3-7<br>4-6<br>7-6<br>4-8<br>5-8<br>6-8	3-5<br>3-6<br>3-7<br>4-6<br>5-8<br>6-8<br>7-8	3-5<br>3-6<br>3-7<br>4-5<br>4-6<br>5-8<br>6-7<br>6-8<br>7-8	3-4<br>3-5<br>3-6<br>3-7<br>4-5<br>4-8<br>6-8<br>7-8	3-4<br>3-6<br>3-7<br>3-8<br>4-5<br>5-8	3-4<br>3-5<br>3-6<br>3-7<br>4-8<br>6-8<br>7-8	3-6<br>3-7<br>4-7<br>5-7<br>6-7<br>7-8
4th Feature Area1	3-4<br>3-5<br>3-6<br>3-7<br>3-8	3-4<br>3-5<br>3-6<br>3-7<br>3-8	3-4<br>3-5<br>3-6<br>3-7	3-4<br>3-5<br>3-6<br>3-7<br>6-8	3-4<br>3-5<br>3-6<br>3-7<br>5-8<br>7-8	3-4<br>3-5<br>3-6<br>3-7<br>3-8	3-4<br>3-5<br>3-6<br>3-7<br>4-8<br>5-8<br>6-8<br>7-8	3-4<br>3-5<br>3-6<br>3-7<br>4-8<br>5-8<br>6-8<br>7-8	3-5<br>3-6<br>3-7<br>4-6<br>4-8<br>5-8<br>6-8<br>7-8	3-5<br>3-6<br>3-7<br>4-6<br>7-6	3-5<br>3-6<br>3-7<br>4-6<br>5-8<br>6-8<br>7-8	3-5<br>3-6<br>3-7<br>4-5<br>4-6<br>5-8<br>6-7<br>6-8	3-4<br>3-5<br>3-6<br>3-7<br>4-8	3-4<br>3-7<br>3-8<br>5-8<br>6-8<br>7-8	3-4<br>3-5<br>3-6<br>3-7<br>4-8<br>6-8<br>7-8	3-6<br>3-7<br>4-7<br>5-7<br>6-7<br>7-8
5th Feature Area2	3-4<br>3-5<br>3-6<br>3-7<br>3-8	3-4<br>3-5<br>3-6<br>3-7<br>3-8	3-4<br>3-5<br>3-6<br>3-7	3-4<br>3-5<br>3-6<br>3-7<br>4-8	3-4<br>3-5<br>3-6<br>3-7<br>4-8<br>5-8<br>6-8<br>7-8	3-4<br>3-5<br>3-6<br>3-7<br>3-8	3-4<br>3-5<br>3-6<br>3-7<br>4-8<br>5-8<br>6-8<br>7-8	3-4<br>3-5<br>3-6<br>3-7<br>4-8<br>5-8<br>6-8<br>7-8	3-5<br>3-6<br>3-7<br>4-6<br>5-8<br>6-8	3-5<br>3-6<br>3-7<br>5-8<br>6-8	3-5<br>3-6<br>3-7<br>4-6<br>5-8<br>6-8<br>7-8	3-5<br>3-6<br>3-7<br>4-5<br>4-6<br>5-8<br>6-8<br>7-8	3-4<br>3-5<br>3-6<br>3-7	3-4<br>3-5<br>3-6<br>3-7<br>3-8<br>4-5	3-4<br>3-6<br>3-7<br>5-7<br>4-8<br>6-8<br>7-8	3-4<br>3-5<br>3-6<br>3-7<br>5-7<br>6-8<br>7-8
6th Feature Ratio1	3-4<br>3-5<br>3-6<br>3-7<br>3-8	3-4<br>3-5<br>3-6<br>3-7<br>3-8	3-4<br>3-5<br>3-6<br>3-7	3-4<br>3-5<br>3-6<br>3-7<br>4-8	3-4<br>3-5<br>3-6<br>3-7<br>4-8<br>5-8<br>6-8<br>7-8	3-4<br>3-5<br>3-6<br>3-7<br>3-8	3-4<br>3-5<br>3-6<br>3-7<br>4-8<br>5-8<br>6-8<br>7-8	3-4<br>3-5<br>3-6<br>3-7<br>4-8<br>5-8<br>6-8<br>7-8	3-5<br>3-6<br>3-7<br>4-6<br>5-8<br>6-8	3-5<br>3-6<br>3-7<br>5-8<br>6-8	3-5<br>3-6<br>3-7<br>4-6<br>5-8<br>6-8<br>7-8	3-5<br>3-6<br>3-7<br>4-5<br>4-6<br>5-8<br>6-8<br>7-8	3-4<br>3-5<br>3-6<br>3-7	3-4<br>3-5<br>3-6<br>3-7<br>3-8<br>4-5	3-4<br>3-6<br>3-7<br>5-7<br>4-8<br>6-8<br>7-8	3-4<br>3-5<br>3-6<br>3-7<br>5-7<br>6-8<br>7-8
7th Feature	4-8	3-6	3-7	**	3-6	3-4	3-8	**	3-5	3-8	**	4-5	5-6	3-4	3-4	**

Ratio2	6-8	3-8<br>4-8<br>5-8<br>6-8<br>7-8	5-7<br>6-7<br>8-7		5-6	3-8<br>4-8<br>5-8<br>6-8<br>7-8			3-8<br>5-6<br>5-7<br>6-8<br>7-8	4-8<br>5-8<br>6-8<br>7-8				3-6<br>3-8<br>4-8<br>5-8<br>6-8<br>7-8	3-5<br>4-6<br>5-6	
8th Feature<br>Ratio3	4-8<br>6-8	3-6<br>3-8<br>4-8<br>5-8<br>6-8<br>7-8	3-7<br>5-7<br>6-7<br>8-7	**	3-6<br>5-6	3-4<br>3-8<br>4-8<br>5-8<br>6-8<br>7-8	3-8	**	3-5<br>3-8<br>5-6<br>5-7<br>6-8<br>7-8	3-8<br>4-8<br>5-8<br>6-8<br>7-8	**	4-5	5-6	3-4<br>3-6<br>3-8<br>4-8<br>5-8<br>6-8<br>7-8	3-4<br>3-5<br>4-6<br>5-6	**
9th Feature<br>NTPower	3-4<br>3-5<br>3-6<br>3-7	3-8<br>4-8<br>5-8<br>6-8<br>7-8	3-4<br>3-5<br>3-6<br>3-7<br>6-8<br>4-8	3-4<br>3-5<br>3-6<br>3-7	3-4<br>3-5<br>3-6<br>3-7<br>4-8<br>5-8<br>7-8	3-4<br>3-5<br>3-8<br>5-6<br>5-7<br>6-8<br>7-8	3-4<br>3-5<br>3-6<br>3-7<br>4-8<br>5-8<br>6-8<br>7-8	3-4<br>3-5<br>3-6<br>3-7<br>4-8<br>5-8<br>6-8<br>7-8	3-4<br>3-5<br>3-6<br>3-7<br>6-8<br>7-8	3-4<br>3-5<br>3-6<br>3-7<br>7-8	3-5<br>3-6<br>3-7<br>4-6<br>5-8<br>6-8	3-5<br>3-6<br>3-7<br>4-5<br>4-6<br>5-8<br>6-7<br>6-8<br>7-8	3-4<br>3-6<br>3-7<br>4-5<br>4-8	3-4	3-4<br>3-6<br>3-7<br>4-5<br>4-8<br>6-8<br>7-8	3-6<br>3-7<br>5-7<br>7-8
10th Feature<br>TPower	3-4<br>3-5<br>3-6<br>3-7<br>3-8	3-4<br>3-5<br>3-6<br>3-7<br>3-8	3-4<br>3-5<br>3-6<br>3-7	3-4<br>3-5<br>3-6<br>3-7	3-4<br>3-5<br>3-6<br>3-7<br>4-8<br>5-8<br>7-8	3-4<br>3-5<br>3-6<br>3-7<br>3-8	3-4<br>3-5<br>3-6<br>3-7<br>4-8<br>5-8<br>6-8<br>7-8	3-4<br>3-5<br>3-6<br>3-7<br>4-8<br>5-8<br>6-8<br>7-8	3-5<br>3-6<br>3-7<br>4-6<br>5-8<br>6-8<br>7-8	3-5<br>3-6<br>3-7<br>4-6<br>7-6<br>5-8<br>6-8	3-5<br>3-6<br>3-7<br>4-6<br>5-8<br>6-8<br>7-8	3-5<br>3-6<br>3-7<br>4-5<br>4-6<br>5-8<br>6-7<br>6-8	3-4<br>3-5<br>3-6<br>3-7<br>4-8<br>7-8	3-4<br>3-6<br>3-7<br>3-8<br>5-8	3-4<br>3-5<br>3-6<br>3-7<br>4-8<br>6-8<br>7-8	3-6<br>3-7<br>5-7<br>7-8

** significance value p>0.05. (3-4 shows that features obtained from EEG signals of GCS 3 and GCS 4 patients are statistically different).
